# Introduction to This Special Issue of *Toxins*: Reduction and Control of Mycotoxins along Entire Food and Feed Chain

**DOI:** 10.3390/toxins15020131

**Published:** 2023-02-05

**Authors:** Alexandra Malachová, Marta Magdalena Sopel, Chibundu N. Ezekiel

**Affiliations:** 1 Austrian Competence Centre for Feed and Food Quality, Safety and Innovation, Technopark 1D, 3430 Tulln, Austria; 2 Wageningen Food Safety Research, Wageningen University and Research, Akkermaalsbos 2, 6708WB Wageningen, The Netherlands; 3 Department of Microbiology, Babcock University, Ilishan Remo 121103, Ogun State, Nigeria

Contamination of food and feed by mycotoxins is considered a significant issue in food and feed safety worldwide. As mycotoxins may enter the food production chain in the early stages, during crop growth, and are able to survive a variety of processing conditions, new approaches and methods for reducing and controlling mycotoxins along the entire food and feed chain are urgently needed.

The contributions selected for this Special Issue of *Toxins* address either novel methods for the prevention of mycotoxin contamination during crop growth and storage or analytical approaches for fast and reliable mycotoxin detection. Eleven manuscripts (nine original research and two review papers) have been compiled for the purpose of this Special Issue.

One strategy for preventing mycotoxin formation is inhibiting the growth of the toxigenic fungi in the field and/or during storage. Current trends involve the application of biological agents rather than synthetic fungicides. A fermented extract of yellow mustard was tested as an antifungal agent against *Fusarium verticillioides* on corn. The obtained results show a reduction of 92.6 % in fumonisin B_1_ levels compared to a control sample [1]. Similarly, mustard’s antifungal properties, which allowed for preventing carcinogenic aflatoxin production during almond storage, were confirmed in another study dealing with the growth inhibition of *Aspergillus flavus* [2]. Moreover, another bioactive substance, allyl isothiocyanate, showed promising results for reducing *A. flavus* populations and aflatoxin B_1_ formation. Like almonds, pistachio nuts are prone to *A. flavus* colonization when stored. The effect of gaseous O_3_ treatment of pistachio nuts on *A. flavus* growth and subsequent aflatoxin production was investigated. Although *A. flavus* colonies were reduced under certain conditions, no effect was seen on aflatoxin B_1_ levels [3].

The safety of biological agents is discussed in the paper by Stranska et al. [4]. Fungal endophytes, on the one hand, promote the growth of the plant and produce bioactive substances needed for better ripening, while on the other, they represent a source of mycotoxins. The toxicogenic potential of fungal endophytes isolated from vineyards was assessed, and most of the isolates were able to produce mycotoxins. Therefore, the effect of the endophyte used for growing *Vitis vinifera* on mycotoxin production should always be properly investigated [4].

The development and optimization of robust and reliable analytical methods for the control of mycotoxins along the entire food and feed supply chain to avoid any harm to human and animal health is critical for all involved parties. Chronic exposure to high levels of mycotoxins through consumption of highly contaminated food is a significant issue in some developing countries. Ezekiel et al. [5] showed high exposure to aflatoxins, citrinin and fumonisins through the consumption of contaminated food and no awareness of mycotoxin issues among households in Nigeria. Thus, interventions aimed at limiting mycotoxins in foods from harvest to storage need to be prioritized. In addition, in order to protect human health, it is necessary to establish routine control of food commodities. A fast, lateral flow device for aflatoxin detection that is robust under sub-Saharan conditions was developed and evaluated in a study published by Cvak et al. [6]. A good correlation was found between the rapid test and confirmatory LC-MS/MS method.

In the European Union, the maximum levels of certain mycotoxins (aflatoxins, deoxynivalenol, zearalenone, fumonisins, ochratoxin A, patulin, citrinin, T-2 and HT-2 toxin, ergot alkaloids) permitted in food- and feedstuff were established by Regulation No. 1881/2006 [7] and Directive 2022/32/EC [8], respectively. Additionally, recommendations for maintaining certain mycotoxins (deoxynivalenol, zearalenone, ochratoxin A, T-2 and HT-2 toxins, fumonisins) below the indicated levels exist [9,10]. Therefore, well-established and validated analytical methods are required for regular control. In order to provide accurate and reliable data, all laboratories involved in the control of mycotoxins participate in the interlaboratory ring trials. A study published by Steiner et al. [11] summarizes the outcome of one voluntary interlaboratory study focused not only on regulated mycotoxins but also so-called emerging mycotoxins recommended for monitoring.

Food supplements might represent a potential health risk to regular consumers, as indicated in a case study by Boško et al. [12]. Milk thistle extract is known for its health benefits due to having antioxidant activity and hepatoprotective effects. However, the outcomes of this study show that it can be contaminated with mycotoxins. Instead of conferring beneficial health effects, regular consumption of such products can be harmful to health [12].

A systematic review written by Farkas et al. [13] summarizes the current approaches and feeding interventions used to control aflatoxins in the diary production chain. A second review paper by Tkaczyk and Jedziniak is focused on biomarker methods used for the assessment of pigs exposed to mycotoxins [14].

Overall, the collected manuscripts bring fresh insights into new approaches for mycotoxin reduction. Furthermore, they stress the need for accurate and reliable analytical tools for mycotoxin detection along the whole production chain in order to keep food and feed safe.
